# Emerging role of artificial intelligence in necrotizing enterocolitis and implementation challenges

**DOI:** 10.1038/s41390-026-05041-0

**Published:** 2026-04-25

**Authors:** Parvathy Krishnan, Vignesh Gunasekaran, William B. Hillegass, François Modave, Parvesh Mohan Garg

**Affiliations:** 1 Tufts Medicine Pediatrics, Division of Newborn Medicine, Boston, MA USA; 2 Tufts University School of Medicine, Boston, MA USA; 3 Department of Pediatrics/Neonatology, West Virginia University School of Medicine, Martinsburg, WV USA; 4 Department of Data Science and Medicine, University of Mississippi Medical Center, Jackson, MS USA; 5 Department of Pediatrics/Neonatology, Atrium Health Wake Forest Baptist, Wake Forest School of Medicine, Winston Salem, NC USA; 6 Department of Biostatistics and Data Science, Wake Forest School of Medicine, Winston-Salem, NC USA; 7 Department of Implementation Science, Wake Forest School of Medicine, Winston-Salem, NC USA

## Abstract

**Abstract:**

Necrotizing enterocolitis (NEC) remains a persistent clinical challenge, with diagnostic strategies largely relying on reactive staging criteria that have not evolved significantly in decades. This commentary synthesizes emerging literature to evaluate the transformative potential of Artificial Intelligence (AI) and Machine Learning (ML) in shifting NEC management toward predictive precision. We review how ML algorithms are redefining risk stratification by integrating multimodal data. AI demonstrates superior utility in automating radiographic diagnosis, analyzing complex data to identify early disease progression, distinguishing medical from surgical phenotypes and providing objective support for difficult intervention decisions. Despite this promise, clinical translation is currently limited by data heterogeneity, small sample sizes, and the black box nature of complex algorithms. Further, the integration of AI methodologies into the clinical IT ecosystem requires careful planning, to assess how such tools affect clinical workflows, how performance changes over time, when they need to be retrained, changed, or shelved, e.g., ML operations (MLOps). We highlight that realizing AI’s potential in the NICU requires a paradigm shift toward multicenter data sharing, the development of explainable AI models, and a rigorous ethical framework to ensure these tools augment rather than obscure clinical judgment.

**Impact:**

- Traditional reliance on Bell’s staging for NEC diagnosis is reactive and lacks the specificity required for early intervention. Artificial Intelligence and Machine Learning offer a transformative approach, demonstrating superior accuracy in risk stratification and surgical prediction by synthesizing complex, multimodal data that elude conventional clinical assessment. Future translational research must prioritize multicenter validation and algorithmic interpretability to safely integrate these predictive tools into real-time neonatal care.

## Commentary

Necrotizing enterocolitis (NEC) is a predominant cause of gastrointestinal morbidity and mortality in premature infants, with mortality rates reaching 50% depending on the disease severity.^1^ NEC has a multifactorial pathogenesis involving immature intestinal immunity, microbial dysbiosis, and ischemic injury. This results in heterogeneous clinical presentations that complicate early detection and management.

Despite nearly five decades of clinical experience, the pathophysiology of NEC remains incompletely understood, and management strategies have not changed significantly. Treatment protocols are still limited to non-specific supportive measures such as total parenteral nutrition, systemic antibiotics, and respiratory support with advanced cases requiring surgical resection.^2^ Diagnosis relies heavily on the Bell’s staging criteria^3,4^ which are fundamentally reactive and lack specificity, particularly in early stages where symptoms are clinically indistinguishable from feeding intolerance or sepsis.^5^ There is also a lack of universal definition, significant inter-observer variability in radiologic interpretation, and the challenge of distinguishing NEC from spontaneous intestinal perforation (SIP).

This high-dimensional complexity makes NEC an ideal candidate for artificial intelligence (AI),^6^ in particular Machine Learning (ML) a subfield of AI. ML is a set of computational methods that learn patterns from biomedical data and make predictions or decisions without being explicitly programmed with fixed rules. Machine learning is commonly divided into two broad approaches: 1) Supervised learning: In this paradigm, we already know the outcome of interest, and we use past examples to teach the model to recognize patterns that predict that outcome. In other words, the model learns from labeled cases. An example of supervised learning is predicting post-operative sepsis using historical surgical cases, where we already know which patients developed sepsis. In this case, the model learns the relationship between patient characteristics and a known clinical outcome of interest. 2) Unsupervised learning is the second paradigm in which there is no predefined outcome. Instead, the goal is to discover structure in the data, typically by identifying groups of patients who share similar characteristics. An example of unsupervised learning is the identification of subtypes of heart failure based on patterns in labs, imaging, and vitals. In this case, the algorithm is not predicting a known label, but rather, it is uncovering patterns that may generate new clinical insights. In other words, supervised learning answers the question “who is at risk,” whereas unsupervised learning answers the question “are there grouping of patients/clinical features that we had not previously identified?”

Unlike traditional statistical models, ML algorithms excel at identifying non-linear patterns and interactions within heterogeneous and multimodal data, integrating variables such as vital sign variability, biomarker profiles, microbiome changes, and subtle radiographic findings.^7,8^ ML finds the model(s) that best fit the data without arbitrary model selection often leading to unrecognized model misspecification. Advanced techniques such as k-fold cross-validation help guard against overfitting the model to the training data to select model(s) with better generalized performance when applied to subsequent clinical data. In this commentary, we synthesize the most recent studies to highlight how AI is actively reshaping the landscape of NEC management, ranging from redefining risk stratification and automating radiographic diagnosis to optimizing the critical timing of surgical intervention.

## Prediction and risk stratification

Traditional risk assessment relies heavily on gestational age (GA) and birth weight (BW). While ML models confirm these as primary predictors, recent studies demonstrate that AI can integrate far more complex variables to enhance predictive power.^9^

AI has demonstrated utility in identifying risk factors that may be missed by traditional observation. For instance, a large study that analyzed 10,353 very low birth weight infants from the Korean Neonatal Network using six ML algorithms identified ambient birth year temperature alongside birth weight and gestational age as a novel predictor (accuracy 0.93, AUC 0.72–0.73).^10^ This highlights AI’s ability to uncover obscure epidemiological links that are typically unrecognized in standard clinical risk models.

Researchers have looked at the utility of ML to predict the risk even prior to birth. Applying ML to fetal Doppler velocimetry in growth-restricted infants, Kim et al. found that normal fetal umbilical artery Dopplers had a 95% specificity for not developing NEC.^11^ This suggests AI could help identify a low-risk cohort.

Most AI models have integrated continuous routine clinical data to predict NEC prior to clinically apparent deterioration. A commonly used ML method, which falls in the tree-based ensemble models category, XGBoost, predicted NEC and other morbidities (AUC 0.73) using standard electronic health record (EHR) data,^12^ using 47 maternal and neonatal features collected in the first two weeks of life. Another tree-based method, The LightGBM model by Mou et al., also demonstrated exceptional performance with an AUC of 0.960, accuracy of 85.8%, and sensitivity of 97% using six clinical features: day of age, gestational age, eosinophil count, hemoglobin, platelet distribution width, and high-sensitivity C-reactive protein.^13^ Meeus et al. utilized continuous physiological monitoring data from 865 preterm infants. Their XGBoost model achieved a median time gain of 10 h prior to clinical diagnosis, offering a critical window for preventative intervention.^14^

Perhaps the most transformative utility of AI lies in its ability to handle very heterogeneous and multimodal data sets to decode the biological black box of NEC pathogenesis, translating complex data into actionable predictions. Multiple instance learning (MIL) analyzing stool microbiota from 3595 samples across 261 infants, achieved 86% sensitivity and 90% specificity to predict NEC onset an average of 8 days in advance, distinguishing cases by specific dysbiosis patterns such as *Proteobacteria* abundance.^15^ Olm et al. further refined this approach using gradient-boosted classifiers to identify specific bacterial replication rates and secondary metabolite gene clusters as predictive features.^16^

## Diagnostic precision

The diagnosis of NEC is often hampered by the subjectivity of abdominal radiograph interpretation. AI can address the subjectivity of Bell’s staging by automating radiographic analysis and combining imaging with clinical metadata.

The interpretation of neonatal abdominal radiographs is notoriously variable, with significant inter-observer disagreement even among experienced clinicians. ML models are now achieving expert-level performance by identifying subtle image features. Deep Learning (DL) is itself a subfield of ML that uses multi-layered neural networks to automatically learn complex patterns from large amounts of data, such as images, waveforms, or even clinical text, without manual feature engineering. A recent DL model analyzing abdominal radiographs from 680 neonates across two centers achieved AUCs of 0.964-0.972 for detecting stage I NEC, outperforming human radiologists in identifying subtle early features.^17^ Similarly, Weller et al. utilized ResNet architectures to distinguish between medical and surgical NEC. Their model achieved an AUC of 0.918, matching the diagnostic accuracy of senior surgical residents. Importantly, it reduced inter-observer variability.^18^

While imaging analysis is crucial, the most powerful diagnostic models integrate radiographic data with clinical biomarkers to mirror the clinician’s thought process. Cui et al. trained a model on over 11,000 laboratory data points and 408 abdominal X-rays.^19^ This model achieved an AUC of 0.91 and an accuracy of 0.94, performance metrics comparable to pediatricians with a decade of clinical experience. Interestingly, this study incorporated gradient-weighted class activation mapping (GradCAM), which visualizes the specific radiographic regions driving the algorithm’s decision, such as fixed loop dilatation or portal venous gas. Such visual proof allows clinicians to trust and verify the AI’s diagnosis, bridging the trust gap.^20^

## Surgical intervention and timing

Determining the necessity and timing of surgical intervention remains a grey zone in NEC management. AI models have attempted to objectively define the surgical phenotype and narrow the window of uncertainty between clinical deterioration and intestinal perforation.

The first challenge lies in distinguishing infants who will respond to medical management. Kim et al. analyzed 16,385 very low birth weight infants from the Korean Neonatal Network using 38 perinatal variables. Their model identified metabolic acidosis, hypotension, and specific CBC trends as dominant predictors of surgical NEC (AUC 0.721).^21^ Similarly, the LightGBM model by Tang et al. identified six independent predictors (CRP, serum lactate, portal venous gas, reduced intestinal motility, white blood cell count, and absolute lymphocyte count) with excellent discrimination (AUC 0.946, sensitivity 0.914, specificity 0.890).^22^ These studies are encouraging as they suggest that ML can create a highly specific severity scoring and surgical prediction integrating metabolic markers with radiographic signs.

AI models have also utilized time-series analysis to offer predictive lead time. Long Short-Term Memory (LSTM) models are a class of DL methods optimized for dealing with sequential data, including vital signs and laboratory trends. The LSTM model with focal loss by Cui et al. used time-series data from 791 neonates to predict surgical need 1–2 days in advance, achieving precision of 0.913 and recall of 0.841 for 1-day predictions.^23^ Qi et al. focused on the imaging aspect of surgical timing. Using Random Forest models to analyze radiomic texture, they identified subtle progression markers such as intestinal wall rigidity and fixed loop patterns before they became clinically obvious. By visualizing these subtle signals of progression, AI can reduce the window of uncertainty between disease progression and perforation.

Despite the theoretical promise of complex algorithms, recent evidence suggests that complexity does not always equal superiority. Interestingly, Li et al. conducted a comparative analysis of five ML models versus a traditional nomogram to predict the optimal timing for surgery. While random forest performed best among the ML models, traditional logistic regression achieved the highest overall accuracy, highlighting that for certain clinical questions, simpler interpretable models may still be superior to complex algorithms.^24^

## Prognosis and severity scoring

AI is also being used to predict disease severity and mortality. The current staging system for NEC is categorical and subjective, often failing to capture the continuum of disease progression. AI models have developed continuous risk scoring that evolves with the clinical course. Ji et al. introduced the use of data-driven algorithms to automate NEC staging. Their linear discriminant analysis (LDA) model integrated clinical and laboratory features such as portal venous gas and metabolic acidosis to stratify infants into low, intermediate, or high risk for disease progression. Unlike Bell’s criteria, this model offered a continuous risk score that predicted progression to surgical disease with an AUC of 0.85, providing a dynamic severity index that could guide escalation of care.^25^ More recently, Nayak et al. developed the HASOFA score (Hyperglycemia, Acidosis, Shock, Organ Failure Assessment). This scoring system achieved an AUC of 0.909 for predicting surgery or death within one week of initial assessment. Crucially, when combined with specific clinical signs like abdominal wall erythema, the predictive accuracy improved to 0.956, offering a potent tool for early intervention for the high-risk group.^26^

Anticipating intestinal perforation is critical for surgical planning, yet clinical signs often appear too late. A recent retrospective analysis utilizing lasso and logistic regression successfully identified independent risk factors for perforation, such as bloody stool, prolonged APTT ( ≥ 50 s), thrombocytopenia, and hypoalbuminemia. The resulting nomogram achieved a robust AUC of 0.838, transforming routine lab values into a predictive tool for one of NEC’s most lethal complications.^27^ Hu et al. extended this predictive capability to imaging, identifying three specific radiomic features on abdominal X-rays as biomarkers for surgical necessity. These texture metrics serve as early warning signs of tissue disintegration before free air becomes visible.^28^

Survival in NEC often comes at the cost of significant morbidity, including intestinal failure. The hybrid prediction model by Bhatt et al. addressed the composite outcome of death or intestinal failure in surgical NEC patients, using four preoperative variables (1 min Apgar score, inotrope use, mean blood pressure, and sepsis) to achieve an AUC of 0.85.^29^ Such models will be helpful to clinicians in identifying infants at highest risk for short gut syndrome and initiate early nutritional rehabilitation strategies.

The precision of prognostic modeling is currently limited by the imprecise definitions of the disease itself. NEC is increasingly conceptualized not as a singular pathology, but as a composite of acquired neonatal intestinal injuries characterized by complex pathophysiology. Recent applications of unsupervised ML are also challenging the fundamental taxonomy of NEC. A recent study identified five distinct clinical phenotypes that defy the conventional binary classification of NEC versus SIP.^30^ These clusters differentiated injuries based on objective physiological and metabolic variances ranging from inflammatory magnitude to gestational maturity. Leveraging AI to disentangle the underlying biological heterogeneity of the disease is a critical prerequisite for future outcomes research.^31^

## Challenges and ethical considerations

Despite the transformative potential of AI in redefining NEC management, the bridge from algorithmic validation to bedside implementation remains with significant technical, infrastructural, and ethical hurdles.

NEC is a relatively rare disease, making it difficult to achieve the massive diverse data required for robust deep learning training. The training of (supervised) ML models refers to the process of showing a computing system many examples of known outcomes so that it learns how to adjust its internal parameters to minimize prediction error and improve future predictions. For DL specifically, this adjustment occurs by slightly modifying millions of internal weights using an optimization method such as gradient descent to reduce a predefined loss function. However, most AI models are trained on small single-center datasets, leading to overfitting.^32^ To avoid overfitting, AI models often require performance assessment on data sets that are vastly different from the data it was training and tested on. This is referred to as external validation. Only a few studies have performed external validation at different centers.^13,15^ Most other models lack this crucial validation step to allow the use of a model across many different health systems. Algorithms that perform exceptionally well on local populations may fail when exposed to the demographic heterogeneity of external institutions. Without multicenter data sharing, AI models risk remaining academically interesting but clinically irrelevant.

Clinical trust must be gained prior to adoption. Complex models like neural networks often lack interpretability. Models often offer high-risk flags without mechanistic justification. Clinicians will often be hesitant to trust a prediction without understanding why the model flagged an infant as high-risk. Explainable AI tools, such as SHAP values, are emerging to map specific features to risk scores.^9^ Low positive prediction of these models is another practical concern. For instance, even high-performing models like XGBoost can generate a positive prediction rate of only 1.3% despite processing hundreds of thousands of data points.^14^ This false alarm can trigger a cascade of defensive measures, including unnecessary sepsis workups, prolonged fasting, and extended central line utilization that carry their own iatrogenic risks. Furthermore, an over-reliance on these automated flags threatens to erode clinical judgment, creating a culture of automation bias, where clinicians treat the algorithmic probability rather than the patient, potentially subjecting low-risk infants to unnecessary aggressive preventative protocols.

Infrastructure requirements for implementation are substantial. The microbiota-based prediction system requires DNA sequencing capabilities and bioinformatics infrastructure.^15^ Continuous monitoring-based models need integration with existing electronic health record systems and real-time data processing capabilities.^14^ Furthermore, building the model is only the beginning. Machine Learning Operations (MLOps) are essential to ensure the model remains accurate, reliable, and safe once it is integrated into real-world clinical workflows. Patient populations change, documentation practices evolve, lab assays shift, and EHR data pipelines get updated. Thus, without continuous monitoring, performance can quietly degrade, a process referred to as “model drift”. MLOps provides structured processes for version control, validation, performance monitoring, bias surveillance, retraining, and governance, so that predictions remain clinically valid over time. In short, MLOps is what turns a promising algorithm into a dependable clinical decision-support tool. No studies have evaluated whether the implementation costs of these AI systems, including infrastructure, training, and maintenance justify the potential benefits in terms of earlier detection or improved outcomes. Ideally, carefully crafted real world evidence studies, such as the methodological standards promulgated by the Observational Health Data Sciences and Informatics Evidence Network will help ascertain whether implementation and integration of AI in the clinical NEC workflow cost-effectively influences practice and improves patient outcomes.^33,34^

The integration of AI necessitates a rigorous ethical framework to prevent algorithmic bias. Models trained on historical data may inadvertently encode systemic biases related to race or socioeconomic status.^35^ Transparency in how these algorithms are validated and monitored is essential to maintain beneficence and non-maleficence. Regulatory pathways for AI-based clinical decision support tools in neonatal care remain unclear, and no NEC prediction models have received FDA clearance or equivalent regulatory approval for clinical use.^36^

AI represents a transformative frontier in NEC research, offering tools to disentangle the complex web of risk factors, biological signals, and clinical signs that have confounded neonatologists for decades. AI holds the potential to enable precision medicine to reduce the morbidity of this devastating disease. However, realizing this potential requires a concerted shift toward multicenter data sharing, rigorous external validation, infrastructure development, and a commitment to ethical implementation.
